# Estimating age specific prevalence and force of infection in Zimbabwe using combined cross-sectional surveys from 2005 to 2015

**DOI:** 10.3389/fepid.2022.1029583

**Published:** 2022-12-06

**Authors:** Rutendo Birri Makota, Eustasius Musenge

**Affiliations:** Division of Epidemiology and Biostatistics, School of Public Health, Faculty of Health Sciences, University of the Witwatersrand, Johannesburg, South Africa

**Keywords:** force of infection, prevalence, Demographic Health Survey (DHS), generalized additive models (GAMs), hazards models

## Abstract

**Objective:**

Age structured sexual mixing patterns have been noted to be associated with HIV prevalence and force of infection. Therefore, this study aimed to estimate the age dependent HIV force of infection using survey cross-sectional data from Zimbabwe.

**Methods:**

We fit generalized additive models namely; linear, semi-parametric, non-parametric and non-proportional hazards models. Using the 2005–06, 2010–11 and 2015 Zimbabwe Demographic Health Surveys data. The Akaike Information Criteria was used to select the best model. The best model was then used to estimate the age dependent HIV prevalence and force-of-infection.

**Results:**

Based on birth year cohort-specific prevalence, the female HIV prevalence reaches the highest peak at around 29 years of age, then declines thereafter. Males have a lower cohort specific prevalence between 15 and 30 years than females. Male cohort-specific prevalence slightly decreases between the ages of 33 and 39, then peaks around the age of 40. The cohort-specific FOI is greater in females than in males throughout all age categories. In addition, the cohort-specific HIV FOI peaked at ages 22 and 40 for females and males, respectively. The observed 18-year age difference between the HIV FOI peaks of males and females.

**Conclusion:**

Our model was appealing because we did not assume that the FOI is stationary over time; however, we used serological survey data to distinguish the FOI's age-and-time effect. The cohort-specific FOI peaked 18 years earlier in females than males, indicative of age-mixing patterns. We recommend interventions that target younger females so as to reduce HIV transmission rates.

## Introduction

There has been a decline in HIV incidence in adults in sub-Saharan (SSA) (1). The prevalence of HIV has increased in those aged 25 and older despite a decrease in HIV incidence due to increasing life expectancy brought on by the advent of antiretroviral treatment (ART) (2–5). A cohort study in Manicaland has highlighted that the cohort of individuals living with HIV in Zimbabwe is aging such that the HIV prevalence is increasing among individuals aged 45 and older and decreasing among younger age groups (3). Due to the high survival rates of those who are HIV infected earlier in life, a shift in the relative share of infections to older individuals has been observed (1). Age-structured sexual mixing patterns have been noted to have an association with HIV force-of-infection (FOI) (6). Force-of-infection (FOI) is defined as the rate at which susceptible individuals acquire an infectious disease, a key parameter that specifies the current state of an epidemic (7). Even though the overall HIV incidence has declined in the era of ART, older cohorts (40–49 years) still account for a growing proportion of HIV incidence (1). Over time, understanding how the HIV FOI rates have shifted across age groups is needed to realign prevention targets.

Given the information about FOI in a population, interventions can be targeted to those at the most significant risk of infection. Subsequently, information on trends in the FOI over time can be used to evaluate the impact of programmes on the rate of new infections so that resources can be directed to the most effective interventions and plan future health care needs. Additionally, understanding the HIV incidence disparities across ages can help prioritize groups where more effort is needed to lower infection rates.

The most direct method of estimating the FOI is using cohort studies. However, these studies are costly; in most cases, representative samples are not attained (8). By contrast, cross-sectional surveys are less expensive, more straightforward, quicker to organize and can be carried out on a mass scale. In addition, serological testing has been more prevalent recently and is now a part of household surveys like the Demographic and Health Survey (DHS), which estimates the prevalence of HIV in the general adult population (9, 10). Given the ease of getting HIV prevalence data from household surveys, mathematical models can help untangle the complicated relationship between HIV FOI and prevalence (11, 12).

One of the modeling approaches to estimating FOI from prevalence data has come from Demography (13, 14). Here, serial cross-sectional population survey estimates of prevalence are used to generate an estimate of the FOI based on the principle that a sample of individuals of age *a* at time *t* is under some conditions representative of a group of individuals aged *a* – *τ* at time *t* – *τ*, where *τ* is the interval between these cross-sectional population surveys (15).

Understanding age-specific differences in the infection rates are essential in predicting the magnitude of the FOI, thus, targeting age-groups for different intervention programs. However, temporal trends and estimates of age-specific FOI have to be recovered from age and time-specific prevalence data (16). In cross-sectional surveys, the information obtained when an individual has been tested is whether that individual has already experienced an infection before the age (*a*) of testing or not. This is commonly known as “current status” data. If the FOI does not change with time, then a single cross-sectional survey of individuals at different ages is sufficient to estimate age-specific infection rates. While this assumption is reasonable for some infections, this assumption is untenable in the case of HIV infection.

This study aimed to estimate the age- and time-dependent prevalence and HIV FOI using current status data from the Zimbabwe Demographic Health Survey (ZDHS) collected in 2005–06, 2010–11 and 2015. This was achieved by developing parametric, semi-parametric and non-parametric models; and determining which models best fit the data.

## Methods

### Zimbabwe survey cross-sectional data

Data from 2005–06, 2010–11, and 2015 ZDHS were used in this analysis and random samples of 10,800, 10,828 and 11,196 households were chosen, respectively. The households were selected using a two-stage cluster sampling method. At the first stage, random samples of 400, 406, and 400 enumeration areas (EAs), were selected for the 2005–06, 2010–11, and 2015 ZDHS, respectively. At the second stage, households were randomly picked using a comprehensive list of all the households in the specified EAs. This made it possible to apply specific weights to EAs in the design (17–19).

#### Outcome variable measurement: HIV testing procedure

Blood samples from all households were taken with the respondent's consent, or that of the respondent's parent or guardian in the case of minors, for HIV testing in the laboratory on females aged 0–49 years and males aged 0–54 years. Finger-prick blood samples were collected on filter paper and taken to a laboratory for analysis. All HIV-positive and 5–10% of the negative tests were retested with a second ELISA after an initial ELISA (enzyme-linked immunosorbent assay) test. A second ELISA or a Western Blot was conducted if the two ELISA tests yielded inconsistent findings. The DHS Data Archive (20) provided the data for this study, which only included people between the ages of 15 and 49. We took into account the sampling design by including survey weights in the analysis.

#### Statistical model

Over the years, methods have been proposed that estimate the age-specific force-of-infection using seroprevalence data (9, 13–16, 21–28). However, these methods are primarily based on an infection acting on a set of susceptible individuals. Given that λ_*A*_(*a*) is the force-of-infection at age *a*, if we assume time homogeneity (stationarity) and lifelong immunity, then the proportion susceptible, *p*_*A*_(*a*) is:


(1) $$p_{A}\left(a\right)=\exp\left[-\int_{0}^{a}{\text{λ}}_{A}\left(z\right)\;dz\right]$$

In reality, time homogeneity assumed in Equation 1 is not feasible as over the years, the introduction of ART, the evolution of cohorts and mutation of the pathogen has resulted in the change of the force-of-infection over time (15). Therefore, the time-specific force of infection λ_*T*_(*t*) can be estimated instead. By integrating exposure experience between the date of birth, *t* − *a*, and the survey date t results in the proportion susceptible *p*_*T*_(*a*) (15):


(2) $$p_{T}\left(a\right)=\exp\left[-\int_{0}^{a}{\text{λ}}_{T}\left(t-a+z\right)\;dz\right]$$

Considering that in this analysis, we deal with three-time points, estimating the force of infection λ_*T*_(*t*), will not be ideal (21). Therefore, while estimating the force-of-infection, especially when working with many time points, it is crucial to model the impacts of age and time effects. The relation between age- and time-specific seroprevalence data and age-time force-of-infection (15), λ(*a, t*), in an individual of age *a* at time *t*, results in the proportion susceptible *p*(*a, t*) (29):


(3) $$p\left(a,t\right)=\exp\left[-\int_{0}^{a}\text{λ}\left(z,\;t-a+z\right)\;dz\right]$$

This paper proposed five models to model the relation between age- and time-specific seroprevalence data and λ(*a, t*) in Equation 3. These models are model 1, representing a parametric model, models 2 and 3, representing semi-parametric models, model 4, representing a non-parametric model, and model 5, representing the non-proportional hazards model. The first is a flexible semi-parametric method suitable for simple exploratory analysis. Then, these methods are applied to age- and time-specific ZDHS HIV seroprevalence data.

The parametric model can capture reasonably complex patterns of age- and time-dependence to determine the degree of curvature required by the data (15). Model 1 constrained the force-of-infection to be positive and is part of the Exponential polynomial (EP) functions which have the following properties (29):


(4) $$\text{λ}\left(a,t\right)=\exp\left(\mu_{0}+\sum \mu_{i}a^{i}+\sum \theta_{j}t^{j}\right),\;i,j=1,2,...$$

where *i* represents the time spent by an individual in age band *i*, and *j* represents the time spent by an individual in time band *j*. We can estimate the age-time dependence of the FOI in Equation 4 by setting all *μ*_*i*_ and *θ*_*j*_ to zero, thus resulting in Equation 5 (15):


(5) $$\begin{array}{l} \text{λ}\left(a,t\right)=\exp\left(\mu a\right) \end{array}$$

Equation 5 represents model 1 and will be referred to in terms of their time/age parameterisation which is model EP1/EP1 being an exponential polynomial of degree 1 in time and degree 1 in age.

Assuming that the FOI changes exponentially with time represented by the following identity (for two calendar times *t*_1_ and *t*_2_) (29) in Equation 6:


(6) $$\begin{array}{l} \text{λ}\left(a,t_{2}\right)=\exp\left(\beta \left(t_{2}-t_{1}\right)\right)\text{λ}\left(a,t_{1}\right) \end{array}$$

We applied the methods of Nagelkerke et al. (12) to estimate age- and time-specific FOI without any assumption about age dependency (16). To be able to assess the cohort experience, a cohort born at calendar time *b* and has age *a* at calendar time *a* + *b*, then the age-specific FOI for that particular cohort is defined by λ_*b*_(*a*) in Equation 7 (29):


(7) $$\begin{array}{l} {\text{λ}}_{b}\left(a\right)=\text{λ}\left(a,a+b\right) \end{array}$$

The proportional hazards assumption in Equation (6) then translates into Equation 8, which leads to models 2 and 3 in this study, which are proportional hazards, semi-parametric models (29).


(8) $$\begin{array}{l} {\text{λ}}_{b}\left(a\right)=\exp\left(\beta \left(b-b_{0}\right)\right){\text{λ}}_{b0}\left(a\right) \end{array}$$

where the hazard at baseline year of birth *b*_0_, is defined by λ_*b*0_(*a*), which can also be expressed as λ(*a, a* + *b*_0_) (29).

The semi-parametric model in Equation (8) can be reformulated as a fully non-parametric proportional hazards model by:


(9) $$\begin{array}{l} \text{λ}\left(a,t_{2}\right)=\exp\left(g\left(t_{2}-t_{1}\right)\right)\text{λ}\left(a,t_{1}\right) \end{array}$$

where *g*(t) is a smooth function with the constraint that *g*(0) = 0.

Substituting Equation (9) in the expression given in Equation (3) leads to:


(10) $$\begin{array}{l} p_{b}\left(a\right)=p\left(a,b+a\right)=\exp\left\{\exp\left(g\left(b-b_{0}\right)\right)\log p_{b0}\left(a\right)\right\} \end{array}$$

where; with λ_*b*0_(*a*) = λ(*a, b*_0_ + *a* ),


$$p_{b0}\left(a\right)=\exp\left\{-\int_{0}^{a}{\text{λ}}_{b0}\left(u\right)du\;\right\}.$$

Equation (10) which is Model 4, may be expressed as a Generalized Additive Model (GAM) for binary data with complementary log-log link function (29) (Equation 11), with π_*b*_(*a*) = 1 − *p*(*a*) representing the proportion of persons infected at age *a* or before from the cohort with year of birth *b* (29):


(11) $$\begin{array}{l} \log\left(-log\left(1-\pi_{b}\left(a\right)\right)\right)=f_{1}\left(a\right)+\;f_{2}\left(b\right), \end{array}$$

where


$$\begin{array}{l} f_{2}\left(b\right)=g\left(b-b_{0}\right), \end{array}$$

and


$$\begin{array}{l} f_{1}\left(a\right)=\log\left(-log\left(x_{b0}\left(a\right)\right)\right). \end{array}$$

The corresponding FOI for this cohort then equals to (29):


(12) $$\begin{array}{l} {\text{λ}}_{b}\left(a\right)\frac{\pi_{b}^{{\;}^{\prime }}\left(a\right)}{1-\pi_{b}\left(a\right)}=\exp\left(f_{2}\left(b\right)\right){\text{λ}}_{b0}\left(a\right), \end{array}$$

with


(13) $$\begin{array}{l} {\text{λ}}_{b0}\left(a\right)\frac{\pi_{b0}^{{\;}^{\prime }}\left(a\right)}{1-\pi_{b0}\left(a\right)}=\frac{d}{da}\exp\left(f_{1}\left(a\right)\right) \end{array}$$

The proportional hazards model translates into an additive *cloglog* model for the seroprevalence status (29), as shown by Equation (11), represented in this study as Model 4. In addition to the year of birth, this GAM technique may be generalized to include additional subject-specific characteristics, such as gender (29).

A non-proportional hazards model (Model 5) was obtained by modeling the interaction of age *a* with year of birth *b*. Taking the GLM model with a two-dimensional smoother (29):


(14) $$\begin{array}{l} \log\left(-log\left(1-\pi_{b}\left(a\right)\right)\right)=f\left(a\;,\;b\right), \end{array}$$

some straightforward calculus shows that:


(15) $$\begin{array}{l} \frac{{\text{λ}}_{b}\left(a\right)}{{\text{λ}}_{b0}\left(a\right)}=\left(\exp\left(f\left(a\;,\;b\right)-f\left(a\;,\;0\right)\right)\right)\left(\frac{\frac{\partial f\left(a\;,\;b\right)}{\partial a}}{\frac{\partial f\left(a\;,\;0\right)}{\partial a}}\right) \end{array}$$

The right-hand side of Equation (15) is no longer independent of age *a*. Note that it reduces to (12) in case *f*(*a*, *b*) =*f*_1_(*a*) + *f*_2_(*b*). The proportional hazards model was tested by comparing a model with a (parametric or non-parametric) (age × year of birth) interaction term with the corresponding model with main effects only (29).

#### Statistical analysis

This study estimated age- and time-dependent prevalence and FOI from serial cross-sectional population survey prevalence data. Our analysis was based on comparing a generalized linear additive model (model 1), generalized semi-parametric additive models (models 2 and 3) and generalized non-parametric additive models (models 4 and 5). All the models were proportional hazards, except for model 5, a non-proportional hazard model. Model 1 adapted Ades and Nokes' (15) exponential polynomial model (EP) of type EP_1_/EP_1_.

Models 2 and 3 were modified from the semi-parametric model of Nagarkerke et al. (16) by employing a spline smoother instead of the isotonic step function for the baseline hazard function λ_*b*0_(*a*). The age-specific FOI, λ_*b*_(*a*), for that particular cohort, was estimated in a semi-parametric way using an iterative MM algorithm with a parametric proportionality factor exp[β (*b* − *b*_0_)] and a non-parametric isotonic stepwise estimate for the baseline hazard λ_*b*0_(*a*) as shown in Equation (6).

Model 4 was reformulated to a fully non-parametric model using the semi-parametric model of Nagarkerke et al. (16) as shown in section Outcome variable measurement: HIV testing procedure. Model 5 was obtained by an interaction of age (a) with the year of birth (b), resulting in a non-proportional hazards model. Models 1–5 are fully explained in section Outcome variable measurement: HIV testing procedure.

#### Model 1–5 specification and goodness-of-fit

We were interested in modeling some response variable *y* (force-of-infection) which follows an exponential family distribution using predictor variables *a* which is age and *b* being the year of birth. Let μ = *E*(*y*), then models 1–5 are:


(16) $$\begin{array}{l} Model\;1\;\left(\;linear\;additive\;model\right):h\left(\mu \right)\\ =\beta_{0}+\beta_{1}a+\beta_{2}b \end{array}$$

(17) $$\begin{array}{l} Model\;2\;\left(semi-parametric\;additive\;model\right):h\left(\mu \right)\\ =\beta_{0}+\beta_{1}b+f_{1}a \end{array}$$

(18) $$\begin{array}{l} Model\;3\;\left(\;semi-parametric\;additive\;model\right):h\left(\mu \right)\\ =\beta_{0}+\beta_{1}a+f_{1}b \end{array}$$

(19) $$\begin{array}{l} Model\;4\;\left(\;non-parametric\;additive\;model\right):h\left(\mu \right)\\ =\beta_{0}+f_{1}a+f_{2}b \end{array}$$

(20) $$\begin{array}{l} Model\;5\;\left(non-proportional\;hazards\;additive\;model\right):h\left(\mu \right)\\ =\beta_{0}+f_{1}\left(a,b\right) \end{array}$$

where *h* is a smooth monotonic “link” function of mean μ and *f*_1_ and *f*_2_ are smooth functions of the covariates considered. In order for these models to be distinguishable, the smooth functions were restricted to have a mean of zero using the gam function. Model specifications for models 1–5 and link function were done using the family argument to generalized additive model (GAM) framework (26) in the R software package *mgcv* (30) with the *gam* function. The Pool-Adjacent-Violators Algorithm (PAVA) application ensured monotonicity in the age dimension. The *mgcv* package's estimation of GAMs used a penalized likelihood method. Finally, we used the Akaike Information Criteria (AIC) to choose the best model for the model comparison.


$$\begin{array}{l} AIC=\underset{goodness\;of\;fit}{\underset{︸}{-2logp\left(a\right)}}+\underset{model\;complexity}{\underset{︸}{2\tau }} \end{array}$$

where *τ* is the effective degrees of freedom (edf). Therefore, if *AIC*_*m*1_ < *AIC*_*m*2_ choose model 1. The effective degrees of freedom (edf) showed the complexity of the model. The 95% confidence interval of the best model was obtained using the R confit.gam package which calculates the point-wise confidence intervals for the smooth terms of a fitted gam model.

## Results

**Figure 1:**
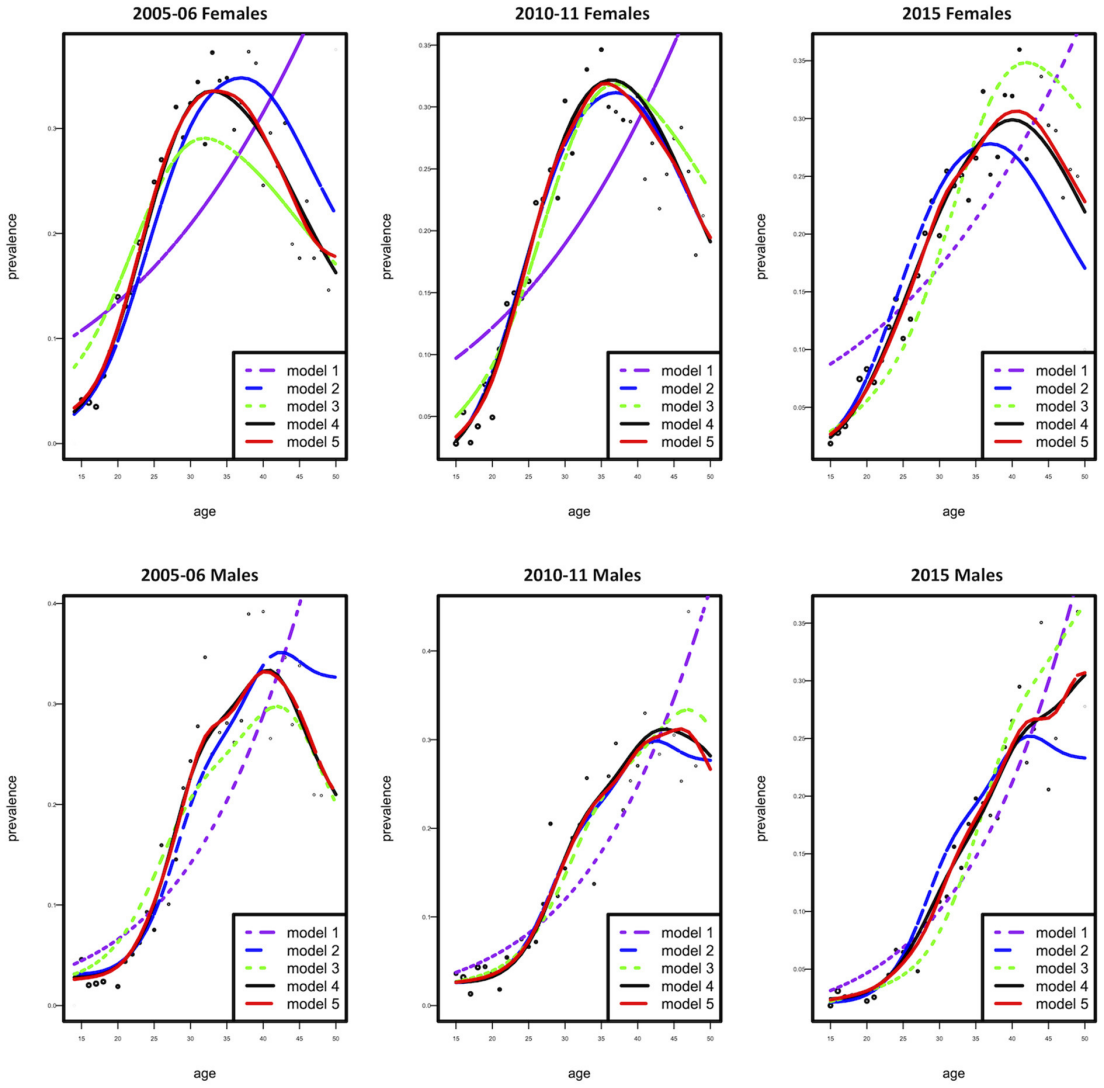
Observed (dotted curve) and fitted prevalence (five solid curves) as a function of age according to females **(top)** and males **(bottom)**.

The dataset was stratified by gender, and all subsequent analysis was done according to the stratification. Generalized non-parametric additive model (model 4) was selected as the best model according to the AIC criteria for females and males (Table 1 and Figure 1). Furthermore, since model 4 was a proportional hazards model, using analysis-of-deviation, the proportionality assumption was determined to be valid for both the female and male models, with *p*-values of 0.71 and 0.50, respectively.

**Table 1:** The Akaike Information Criterion (AIC), Schwarz's Bayesian Criterion (BIC), and effective degrees of freedom (edf) for completely parametric (model 1), semi-parametric (models 2 and 3), and non-parametric (models 4 and 5) models.

**Model components**	**Females**					**Males**				
	**edf**	**AIC**	**Rank**	**BIC**	**Rank**	**edf**	**AIC**	**Rank**	**BIC**	**Rank**
Model 1: *a* + *b*	3	22,571.69	5	22,596.00	5	3	12,196.91	5	12,220.32	5
Model 2: *f*(*a*) + *b*	6.99	21,841.76	3	21,897.11	2	8.25	11,968.76	3	12,033.11	1
Model 3: *a* + *f*(*b*)	6.61	21,945.97	4	21,999.54	4	8.03	12,003.26	4	12,065.93	4
Model 4: *f*(*a*) + *f*(*b*)	9.93	21,779.61	1	21,860.10	1	12.5	11,938.44	1	12,035.99	2
Model 5: *f*(*a, b*)	15.7	21,785.40	2	21,912.22	3	15.5	11,939.81	2	12,060.90	3

a, age; b, year of birth; f, smooth function.

**Figure 2:**
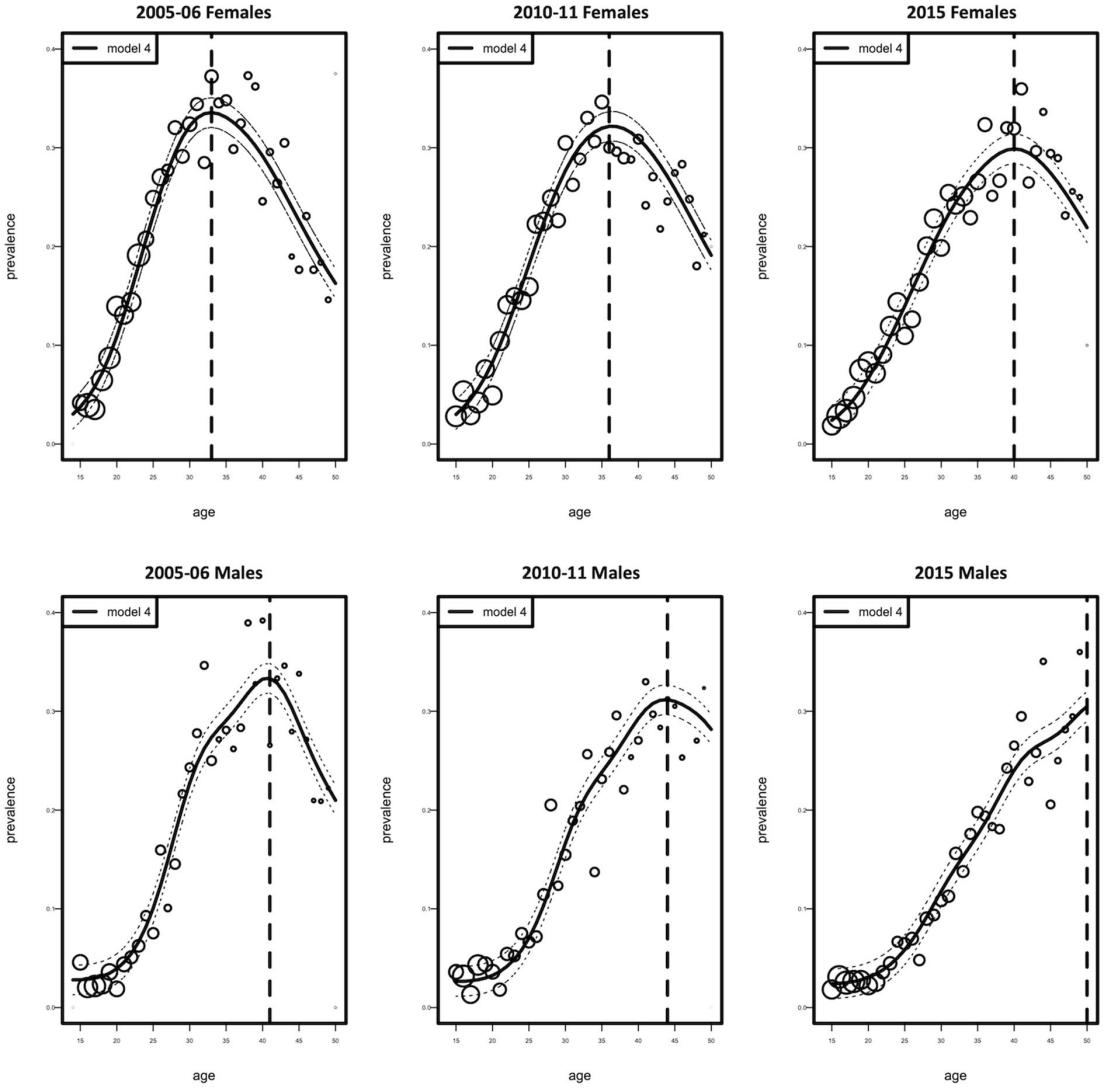
Observed (circles) and fitted (solid curve with 95% Confidence Interval, dotted curve) HIV prevalence as a function of age according to females and males. Circles represent the estimated prevalence by age. Their size is proportional to the number of individuals.

Figure 2 shows the age-specific fitted and observed HIV prevalence estimates for the three calendar periods 2005–06, 2010–11, and 2015, for both females and males. Model 4 showed a good model fit for the observed data. Comparing the fitted HIV prevalence model, we detect a change of high HIV prevalence among 33-year-old females in 2005–06, 36-year-old females in 2010–11, and 40–year-old females in 2015. In 2005–06, the prevalence of HIV was highest among 42-year-old males; however, in 2010–11, the prevalence declined in the same age group. In 2015, the prevalence of HIV steadily rose with age among males. In general, the prevalence of HIV was greater among females than males over all three time periods.

**Figure 3:**
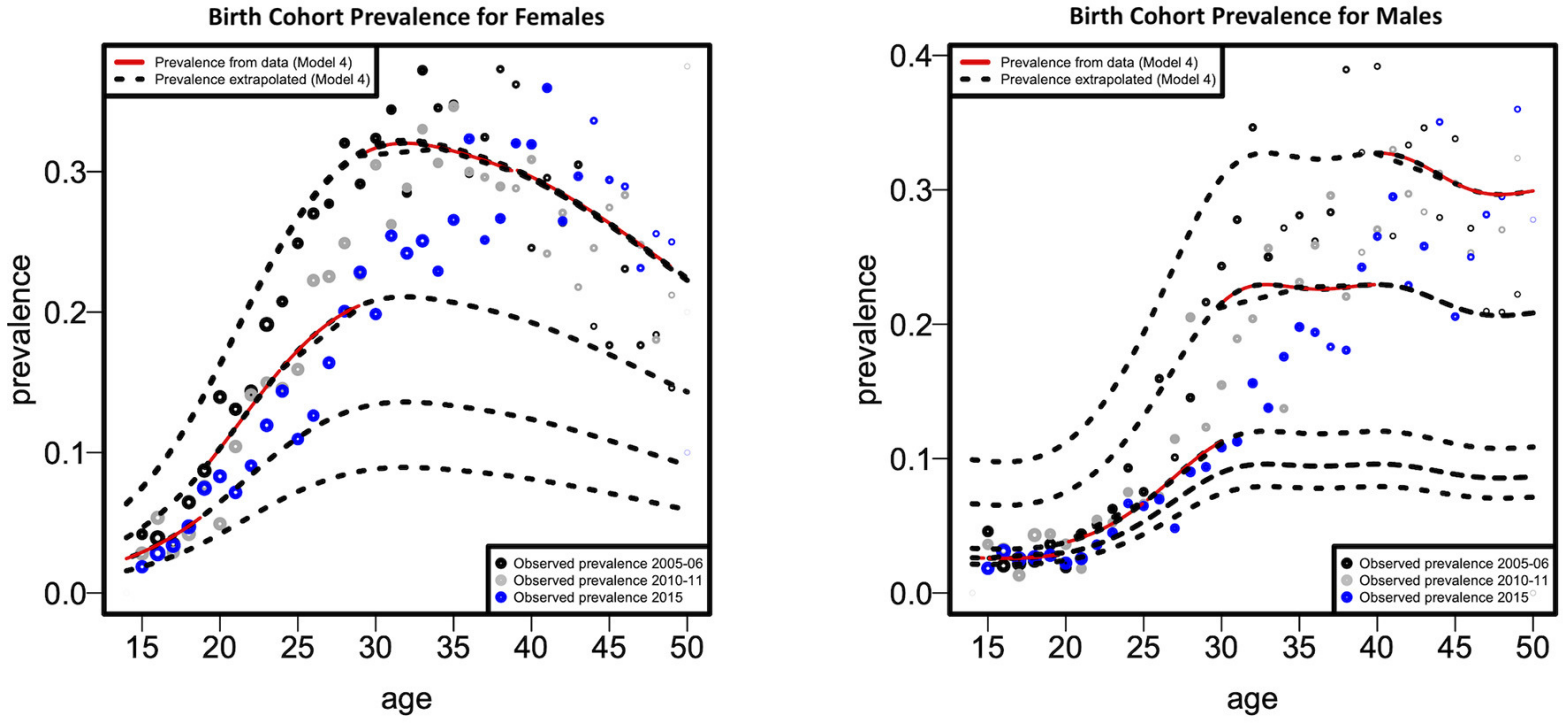
Fitted cohort-specific prevalence curves as a function of age according to females and males.

Figure 3 illustrates the fitted cohort-specific prevalence curves for females and males as a function of age, according to the best model (model 4). Prevalence was estimated for each birth cohort for all survey years combined. The solid red component of each prevalence curve corresponds to the proportion of the age range (birth cohort) for which data were available, while the dotted portion of each curve was extrapolated using the model. Figure 3 demonstrates that data for both models were available for the age range considered in this study. Based on cohort-specific prevalence, the female HIV prevalence reaches the highest peak at around 29 years of age, then declines up to the age of 49 years (these values can be observed by examining the red lines/curves). Males, have a lower cohort-specific prevalence between 15 and 30 years than females. Male cohort-specific prevalence slightly decreases between the ages of 33 and 39. The highest peak of HIV prevalence for males is around the age of 40.

**Figure 4:**
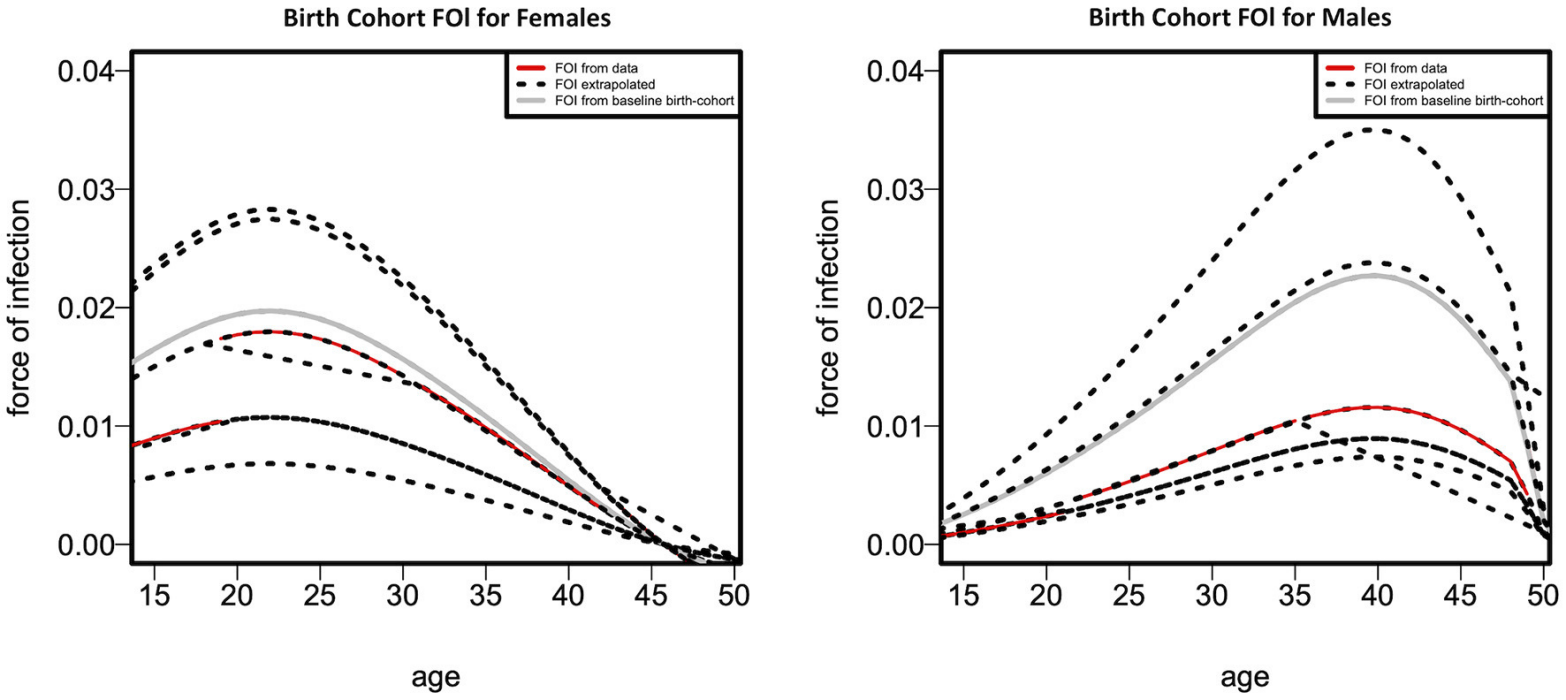
Fitted cohort-specific force-of-infection curves as a function of age according to females **(left panel)** and males **(right panel)**.

Figure 4 depicts the fitted cohort-specific FOI curves as a function of age for females and males, as predicted by Model 4. The FOI was estimated for every birth cohort over the combined survey years. The gray FOI curve displays the baseline FOI λ*b*_0_ (*a*) for an assumed *b*_0_ = 1,955, 1,955 being the minimum time (birth year) in the cohort. The black dotted curves are generated from the proportionality factor exp(*g*(*b* – *b*_0_)) in Equation 11 resulting in the red curves. Figure 4 indicates that, where data is available, the cohort-specific FOI is greater in females than males throughout all age categories, as indicated by the red curve. The cohort-specific HIV FOI peaked at ages 22 and 40, respectively, for females and males, demonstrating more than 15-year age difference between the HIV FOI peaks of males and females. Nevertheless, based on the extrapolated cohort-specific FOI, males tend to have a greater FOI than females in older age groups.

## Discussion

This study estimated the age- and time-dependent HIV prevalence and FOI. Our model was appealing because we did not assume that the FOI is stationary over time; however, we used serological survey data to distinguish the FOI's age-and-time effect. It has been reported that the estimation of HIV FOI might lead to over-interpretation of results because of selection bias, partially due to lower consent rates (27, 31). However, recent work has confirmed that non-participation in surveys does not result in significant biases in estimating cross-sectional HIV prevalence (2, 27).

Generally, HIV prevalence decreased for both males and females over the years, with HIV prevalence higher in females than males. Of interest, we observed the peak HIV prevalence for each successive survey year was lower but shifted with an increase in age, which can be explained by either that people are now being infected at older ages or that these are people who were diagnosed with HIV many years after being infected. Unfortunately, data on HIV testing patterns in different age groups are unavailable, and the observed age increase may have resulted from increased testing in cohorts born earlier. In another school of thought, it is believed that due to the scale-up of antiretroviral treatment, there has been an increase in life expectancy; therefore, the prevalence in the population for the cohorts born earlier will increase (32). However, the shift toward increased HIV prevalence in older birth cohorts is typical of populations of people living with HIV (PLHIV) across the world (33). Our model, especially for males, projects similar age profiles (Figure 1).

A sharp increase in cohort-specific HIV prevalence is observed in our results to occur approximately a decade apart in females (at 29 years) than males (at 40 years). Cohort-specific FOI peaks ~15 years earlier in females (at 22 years) than males (at 40 years). These findings confirm the issue of age-mixing patterns which can be corroborated by other studies (6, 31, 34). A study in Zimbabwe found that age-disparate sexual relationships are associated with increased HIV incidence among young women, mainly when partners are ten or more years older (35). In a 2005 national household survey done in South Africa, it was determined that younger females had partners who were at least 5 years older than them, and it was hypothesized that these connections were linked to an increased HIV risk (36). Gregson et al. offered empirical evidence that age-disparate sexual interactions were responsible for the observed variations in the epidemiological patterns of young women and men in rural Zimbabwe (37). Therefore, age-disparate sexual relationships expose individuals to an increased risk of HIV infection. It has also been noted that age-mixing in sexual relationships is likely to reduce the younger person's ability to negotiate safe sex successfully (38). Increasing female education regarding factors that increase HIV transmission rates might reduce age-disparate relationships, thereby decreasing infection rates in this age group. Pettifor et al. observed that education might lower young women's susceptibility to HIV infection, suggesting that interventions that target structural and partner-level risk factors, such as keeping young women in school, are necessary to lessen this vulnerability (39). This notion is supported by a study that found that higher female education was associated with a lower occurrence of age-disparate relationships (40).

A significant limitation of our study was that we did not address the issue of differential selection. This is when disease-related mortality affects the interpretation of observed serological profiles. Instead, we assumed that the excess mortality of individuals testing positive was negligible. For obvious reasons, this is a crude assumption, and other authors have proposed models which address this issue (13, 21, 26). However, our main advantage was combining three cross-sectional surveys from different time points, thereby jointly modeling age and epidemic effects. Since these surveys are conducted after every 5 years, the next one was scheduled for 2020 but because of COVID, no recent DHS survey has been conducted for Zimbabwe.

Combined cross-sectional surveys should be used to evaluate control measures and monitor the trends in HIV FOI. The same age groups should also be adopted for each successive survey, assuming that the temporal trend in the FOI can be monitored from the changes in prevalence. Future work would significantly be important to include differential selection and refine model estimates within a Bayesian framework by integrating other data sources.

## Data availability statement

Publicly available datasets were analyzed in this study. This data can be found here: Demographic Health Survey Program website [https://dhsprogram.com].

## Ethics statement

The studies involving human participants were reviewed and approved by the Human Research Ethics Committee (Medical) of the University of Witwatersrand (No. M151154). Written informed consent to participate in this study was provided by the participants' legal guardian/next of kin.

## Author contributions

RB and EM conceived of the presented idea and verified the analytical methods and models. RB developed the theory and performed the data analysis. EM supervised the findings of this work. Both authors discussed the results and contributed to the final manuscript.

## Funding

This work was supported through the Developing Excellence in Leadership Training and Science Africa (DELTA) initiative. The DELTA's Africa Initiative is an independent funding scheme of the African Academy of Sciences (AAS)'s Alliance for Accelerating Excellence in Science in Africa (AESA) and supported by the New Partnership for Africa's Development Planning and Coordinating (NEPAD) Agency with funding from the Wellcome Trust [Grant 107754/Z/15/Z—DELTAS Africa Sub-Saharan Africa Consortium for Advanced Biostatistics (SSACAB) programme] and the United Kingdom (UK) government.

## Conflict of interest

The authors declare that the research was conducted in the absence of any commercial or financial relationships that could be construed as a potential conflict of interest.

## Publisher's note

All claims expressed in this article are solely those of the authors and do not necessarily represent those of their affiliated organizations, or those of the publisher, the editors and the reviewers. Any product that may be evaluated in this article, or claim that may be made by its manufacturer, is not guaranteed or endorsed by the publisher.

## Author disclaimer

The views expressed in this publication are those of the author(s) and not necessarily those of AAS, NEPAD Agency, Wellcome Trust or the UK government.
